# Nitrogen Fertilizer Type and Genotype as Drivers of P Acquisition and Rhizosphere Microbiota Assembly in Juvenile Maize Plants

**DOI:** 10.3390/plants12030544

**Published:** 2023-01-25

**Authors:** Melissa Mang, Niels Julian Maywald, Xuelian Li, Uwe Ludewig, Davide Francioli

**Affiliations:** Institute of Crop Science, Department of Nutritional Crop Physiology, University of Hohenheim, 70599 Stuttgart, Germany

**Keywords:** ammonium, nitrate, phosphorous acquisition, plant–microbe interactions, arbuscular mycorrhizal fungi

## Abstract

Phosphorus (P) is an essential nutrient for plant growth and development, as well as an important factor limiting sustainable maize production. Targeted nitrogen (N) fertilization in the form of ammonium has been shown to positively affect Pi uptake under P-deficient conditions compared to nitrate. Nevertheless, its profound effects on root traits, P uptake, and soil microbial composition are still largely unknown. In this study, two maize genotypes F160 and F7 with different P sensitivity were used to investigate phosphorus-related root traits such as root hair length, root diameter, AMF association, and multiple P efficiencies under P limitation when fertilized either with ammonium or nitrate. Ammonium application improved phosphorous acquisition efficiency in the F7 genotype but not in F160, suggesting that the genotype plays an important role in how a particular N form affects P uptake in maize. Additionally, metabarcoding data showed that young maize roots were able to promote distinct microbial taxa, such as arbuscular mycorrhizal fungi, when fertilized with ammonium. Overall, the results suggest that the form of chemical nitrogen fertilizer can be instrumental in selecting beneficial microbial communities associated with phosphorus uptake and maize plant fitness.

## 1. Introduction

Phosphorus (P), which is immobile in soil and poorly available to plants, is a crucial factor in maize production. This is due to the fact that plant-available phosphates (P_i_) in the soil represent less than 0.1% of total soil P, because organic and inorganic P is chemically bound to calcium, magnesium, iron, or aluminium oxides/hydroxides in a pH-dependent manner [1,2,3,4]. However, plants have developed a variety of strategies to cope with poor P availability and mobilize P in soil. In general, most plants increase their root/shoot ratio and increase the soil–root interface by various means. Photoassimilates are translocated from the shoot to the root to increase root branching in the topsoil and enhance root hair density and length, as well as lateral root length [5,6,7]. In addition, energetic costs are minimized by reducing root transpiration through the formation of cortical aerenchyma [7,8]. For P_i_ mobilization in the rhizosphere, plant roots exudate sugars, organic anions, amino acids, secondary metabolites, and protons into the rhizosphere [9,10,11]. On the one hand, this helps plants to acidify the rhizosphere to solubilize calcium phosphates, but on the other hand, this promotes associations with soil microorganisms that potentially enhance P availability and increase plant growth [12]. A well-known example in this context is mycorrhizal colonization of maize roots, which promotes P_i_ acquisition [13,14]. In addition, maize seedlings grown under low-phosphorous conditions show greater reliance on mycorrhizae than on root hairs [15], highlighting the important role of beneficial microorganisms in maize P acquisition. Interestingly, these adaptations to P deficiency are strongly dependent on the maize genotype. For example, under low-P conditions, several recently released maize elite flint lines developed a smaller root system with shorter root hairs compared to older founder flint lines at juvenile stages [16]. In addition, maize inbred lines differed greatly in their responsiveness to arbuscular mycorrhizal fungi under low-phosphorus conditions [17,18]. Among a panel of flint lines, a recently developed doubled haploid landrace displayed the highest mycorrization, showing the potential of old material for breeding of positive symbiotic traits into modern varieties [19]. Options to enhance plant growth in the presence of limited P availability include inoculation with plant-growth-promoting rhizobacteria or mycorrhizal fungi. Furthermore, the availability of different nitrogen forms may affect the presence or activity of beneficial soil microbes in the context of P_i_ acquisition. Such mechanisms may, however, only become relevant at later growth stages, as young developing maize plants initially exclusively rely on nutrient remobilization from the seeds, especially under low P_i_ availability [16], as maize initially has generally a rather small root for supplying the large shoot. 

Nitrogen fertilization improves nutrient utilization and uptake in low-P_i_ soils by promoting root growth and exudation and by altering rhizosphere processes [20,21,22]. In particular, nitrogen in the form of ammonium leads to rhizosphere acidification, which is frequently associated with positive changes in rhizosphere chemistry, increasing P_i_ availability and uptake [23]. Clearly, maize plants show distinct sugar and amino acid metabolism and altered physiology when they are supplied by either nitrogen form or mixed nitrogen sources [24]. Nitrogen then affects exudate profiles, and additionally, ammonium as a nitrogen source is known to affect root physiology and architecture in a different way than nitrate. For example, *Arabidopsis* lateral roots show high plasticity to different N forms. While nitrate stimulates lateral root elongation, ammonium stimulates both primary and lateral root development [25,26,27,28,29]. Ammonium nutrition has also been found to increase the presence and abundance of putative beneficial soil microbial organisms, which is of particular interest with respect to plant-growth-promoting and P-solubilizing bacteria and fungi [30]. In addition, the ability of P-solubilizing microorganisms to increase phosphate availability depends on the nitrogen source, with greater P solubilization found in the presence of ammonium salts compared to nitrate [31]. 

In this study, two different chemical nitrogen fertilizers (ammonium and nitrate) were used to determine their effects on plant fitness parameters, root characteristics, P efficiency, and the associated microbiomes of two contrasting maize lines from the heterotic flint pool (old F7 and modern F160) during their early growth stage. Screening of various flint lines at the juvenile stage revealed varying P_i_ responsiveness among the different maize lines, with the juvenile biomass of F7 being twice that of F160 at sufficient P supply, while the biomass was similar at low-P conditions [16]. It was tested whether such a result depended on the form of nitrogen supplied to the soil, and it was found that in low-P conditions, different chemical forms of nitrogen (ammonium and nitrate) affected plant fitness at early stages. Thus, it can be hypothesized that (i) ammonium application would improve phosphorous acquisition efficiency (PAE) compared to nitrate fertilization, with stronger effects expected for the P-responsive genotype F7 than the non-P-responsive genotype F160. Because the soil microbial community plays critical roles for plant nutrition and crop fitness, amplicon sequencing was used to characterize the bacterial and fungal communities associated with the rhizosphere of the two maize genotypes, as well as the bare soil. It was further hypothesized that (ii) the presence of juvenile maize roots would shape the rhizosphere microbial communities in a general and genotype-specific way and that (iii) nitrogen fertilizer type promotes distinct microbial taxa related to maize nutrition and fitness.

## 2. Results

### 2.1. Low P_i_ Availability Reduces Maize Biomass and Affects the General Nutritional Status

**Figure 1:**
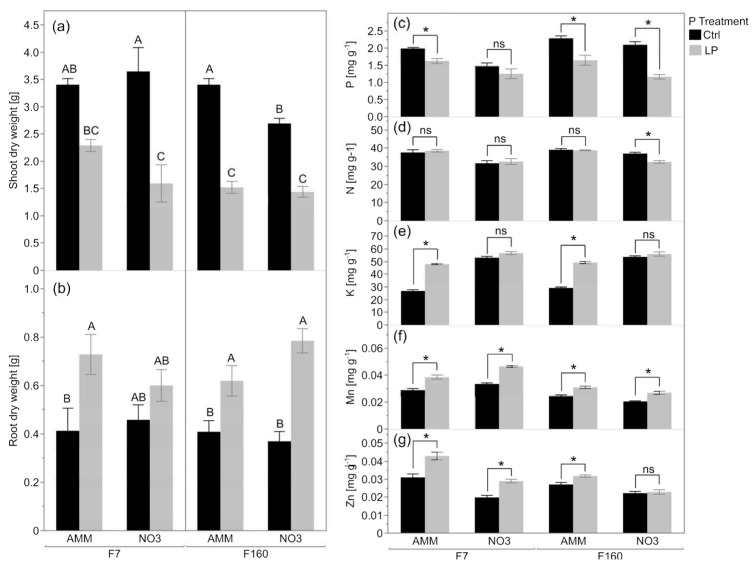
Plant fitness parameters of two genotypes (F160 and F7) grown under controlled (Ctrl, black bars) or low-phosphorus conditions (LP, grey bars), fertilized with either ammonium (AMM) or nitrate (NO3). Shoot (**a**) and root (**b**) dry weight [g], shoot phosphorus concentration (**c**), shoot nitrogen concentration (**d**), shoot potassium concentration (**e**), shoot manganese concentration (**f**), and shoot zinc concentration (**g**) [mg g^−1^] of four-week-old maize seedlings. Data represent mean values of five biological replicates ± SE. In (**a**,**b**), significant differences between N and P treatment within a genotype are indicated by different capital letters according to Tukey’s HSD at *p* < 0.05. In (**c**–**g**), significant differences in P treatment according to Tukey’s HSD at *p* < 0.05 are shown as asterisk (*). No significant differences are indicated by ns.

Maize plants were grown for four weeks in a soil–sand mix (made from fresh topsoil with a pH 6.89) that was amended with all required nutrients, except for P. Phosphorus was either applied for optimal growth or at a reduced level. Consequently, in the low-P conditions, plants suffered P deficiency, which was associated with a reduction in shoot biomass compared to the fully fertilized control plants. With nitrate as the N fertilizer form, genotype F7 showed a 56% decrease in shoot biomass when P supply was limited (Figure 1a). No significant changes in F7 shoot biomass were found in the corresponding ammonium treatment under low-P conditions. It was observed that genotype F160 reduced shoot biomass and increased root biomass under P limitation regardless of N fertilizer form (Figure 1a,b). In F7, however, an increase in root biomass due to low P availability was observed exclusively with ammonium fertilization. In general, the P concentration in the shoots of both genotypes was reduced in the low-P treatment compared to the P-fertilized control, with the exception of F7 plants with nitrate fertilization (Figure 1c). Only the genotype F160 when fertilized with nitrate had its leaf N content decreased by about 13.5% under low P, while no differences were measured in all the other treatments (Figure 1d). Maize plants fertilized with ammonium showed an increase in leaf K concentration under low P compared to the respective control (Figure 1e). Nitrate-fertilized plants showed no differences in K concentration. Mn concentrations in leaves increased in low P compared to the control (Figure 1f), and leaf Zn showed a similar trend, although no differences were observed between low P and the control in shoots of genotype F160 when fertilized with nitrate (Figure 1g).

Next, the effects of different chemical nitrogen forms, P levels, genotype, and their interaction on P use efficiency (PUE), P utilization efficiency (PUtE) and P acquisition efficiency (PAE) of juvenile maize plants were investigated. This formalism was used despite the fact that at this stage, the maize plants have acquired only little more than the P stored in the seed from the soil, suggesting that a large fraction of the P is remobilized in any case P from seed resources [16]. In this context, the P level and N treatments affected all three P efficiency measures, while PUE (F = 7.2; *p* = 0.0114) and PUtE (F = 5.2; *p* = 0.0292) were also influenced by the genotype (Table 1). Phosphorous uptake by maize roots was mainly influenced by the chemical N fertilizer form (21.1; *p* < 0.001) and by the P level (F = 5.8; *p* = 0.0221). A significant interaction of genotype and P treatment on internal P_i_ utilization (F = 6.4; *p* = 0.0170) was found, while the interaction of all three main effects affected PUE (F = 5.7; *p* = 0.0232). These results suggested that maize plants grown in P-limited soil suffered from severe P deficiency compared to the control, which translated into either a reduction in the shoot biomass or the shoot P concentration, or both. Mechanistically important plant and soil key factors related to the effect of the different nitrogen form on P nutrition were then studied in detail at low P availability.

### 2.2. Effect of Genotype and N Nutrition on Plant Attributes in P-Limited Soil

Various plant characteristics were measured, together with rhizosphere pH, to determine the effects of different N forms on the root system and leaf nutrient status of both maize genotypes under P limitation (Table 2). Root/shoot ratio, total root length (TRL), specific root length (SRL), and fine root length (FRL) were not affected by N-form or genotype. Plants of genotype F7 fertilized with ammonium had the largest average root diameter (RD) compared to nitrate-fertilized plants of both genotypes. Ammonium-fertilized plants of genotype F160 showed the highest percentage of root length colonized by arbuscular mycorrhizal fungi (M), while all other plants showed no differences in mycorrhization. Nitrate-fertilized F160 plants exhibited longer root hair length (RHL) compared to ammonium-fertilized plants, while F7 plants had no differences in RHL between the N treatments. Rhizosphere pH was not different with respect to the chemical nitrogen forms in F7, but a decrease in rhizosphere pH from 7.27 to 7.04 was observed in F160 with ammonium fertilization.

**Figure 2:**
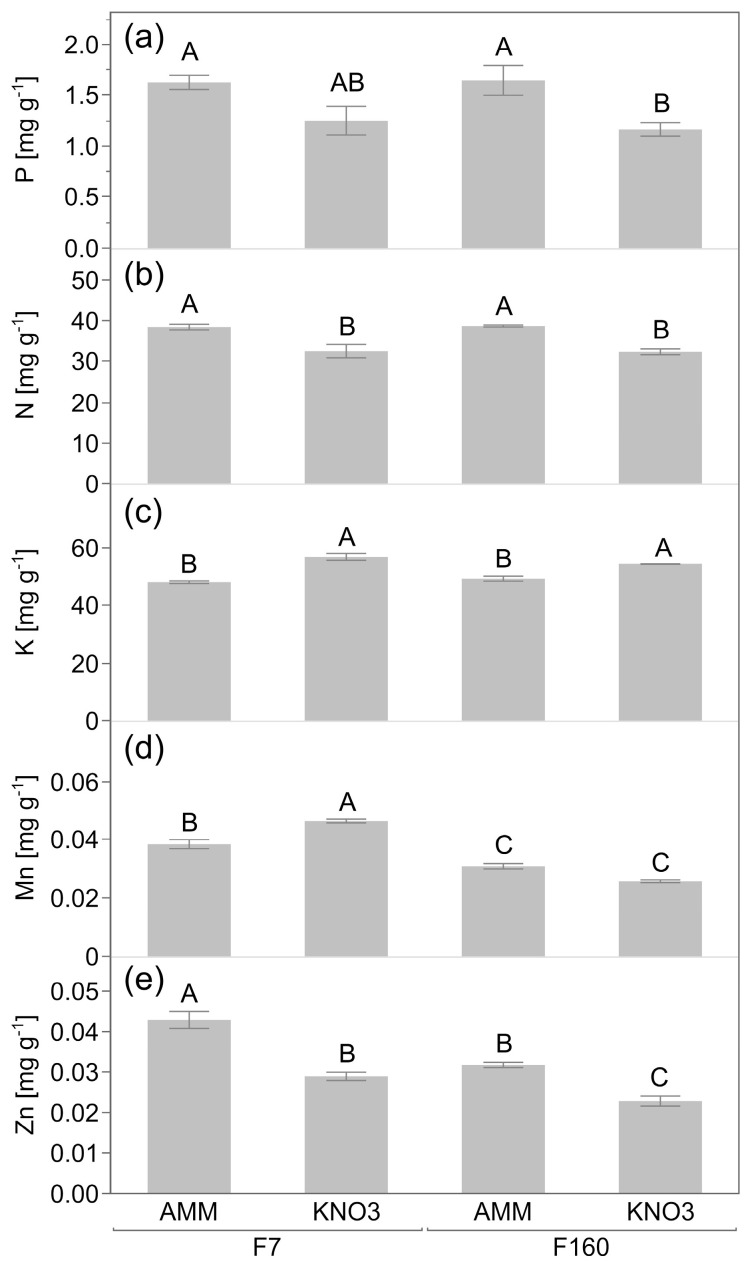
Leaf nutrient concentration of two genotypes (F160 and F7) grown under low-phosphorus conditions (LP) fertilized with either ammonium (AMM) or nitrate (NO3). Shoot phosphorus concentration (**a**), shoot nitrogen concentration (**b**), shoot potassium concentration (**c**), shoot manganese concentration (**d**), and shoot zinc concentration (**e**) [mg g^−1^] of four-week-old maize seedlings. Data represent mean values of five biological replicates ± SE. Different capital letters indicate significant differences at *p* < 0.05 according to Tukey’s HSD.

The different chemical N fertilizer form also affected the plant nutritional status for P, N, and other elements. The leaf N concentration was significantly (*p* < 0.05) increased by around 16.5 % with ammonium compared to nitrate (Figure 2b). Similarly, the shoot P concentration was significantly (*p* < 0.05) higher in the ammonium treatment compared to nitrate for both genotypes (Figure 2a). The opposite trend was observed for leaf K concentration (Figure 2c). K was significantly (*p* < 0.05) higher in nitrate-fertilized plants. Leaf Zn and Mn concentrations varied depending on maize genotype and fertilizer. Leaf Zn was always higher in the ammonium treatment than with nitrate, with the highest concentration found in F7 (Figure 2e). On the contrary, leaf Mn was significantly (*p* < 0.05) higher with nitrate in F7, while this micronutrient did not differ in F160 between the two N treatments (Figure 2d).

**Figure 3:**
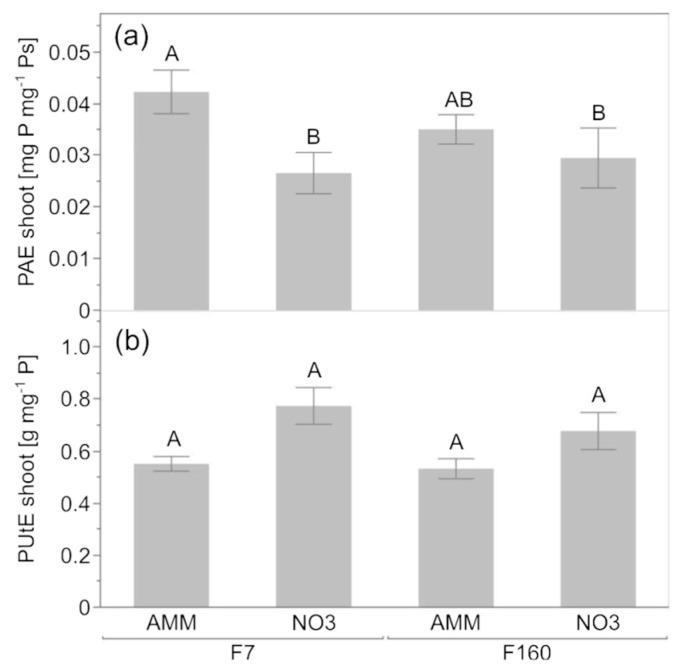
Phosphorus acquisition (**a**) and utilization (**b**) efficiency in maize seedlings of two genotypes (F160 and F7) grown under low-phosphorus conditions fertilized with either ammonium (AMM) or nitrate (NO3). Data represent mean values of five biological replicates ± SE. Different capital letters indicate significant differences at *p* < 0.05 according to Tukey’s HSD.

Lastly, the PUtE and PAE were formally calculated to pinpoint how different forms of N affected P_i_ uptake, translocation, and utilization in the early growth stage of maize. In ammonium-fertilized F7, the PAE increased by 42.6% compared to nitrate (Figure 3a), but the PAE of F160 was not different among the two N treatments. PUtE was not affected by genotype or N fertilizer form (Figure 3b).

### 2.3. Microbial Composition of the Anlayzed Samples

**Figure 4:**
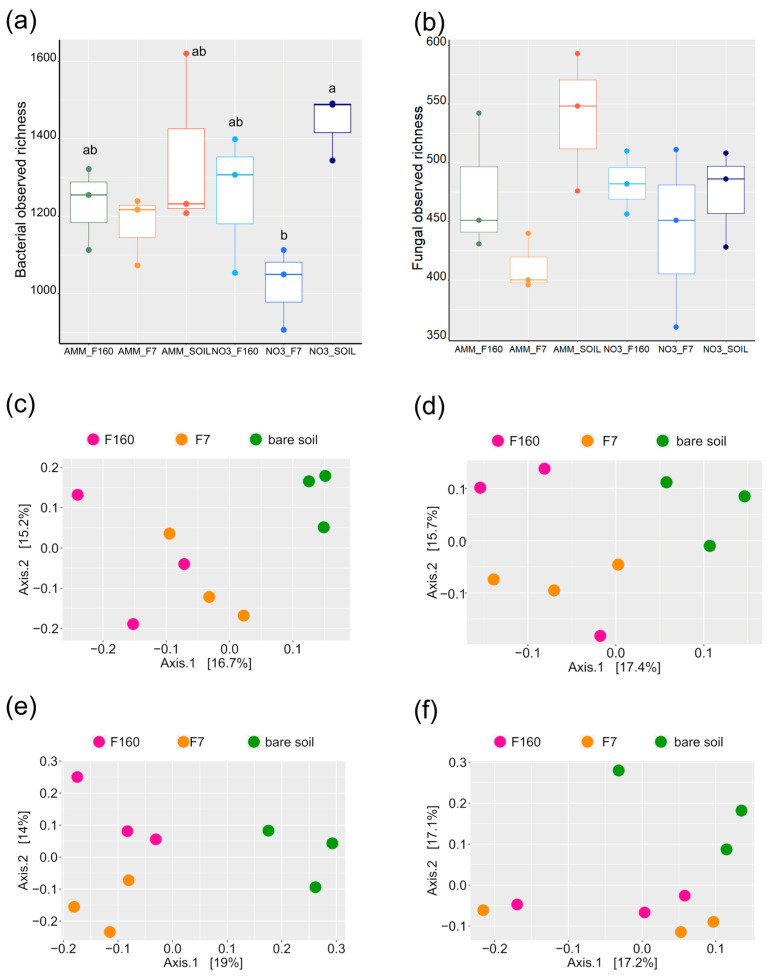
Box plots of the observed richness of (**a**) bacterial and (**b**) fungal community in the experimental treatments studied. The experimental treatments included fertilization with ammonium (AMM) or nitrate (NO3) and addition of genotype F160 or F7 or no plant (SOIL), resulting in the abbreviations used (AMM_F160; AMM_F7; AMM_SOIL; NO3_F160; NO3_F7; NO3_SOIL). Principal coordinates analysis of the bacterial (**c**) and fungal (**d**) communities in the ammonium treatment of genotype F160 or F7 or no plant (SOIL). Principal coordinates analysis of the bacterial (**e**) and fungal (**f**) communities in the nitrate treatment of genotype F160 or F7 or no plant (SOIL). In the boxplots, different letters indicate significant differences based on Tukey’s HSD test, *p* < 0.05.

A total of 1,153,878 16S bacterial and 709,642 ITS (internal transcribed spacer) fungal high-quality reads were obtained from the 18 samples analyzed, which were clustered into 9082 bacterial and 2393 fungal ASVs (amplicon sequence variants). In total, bacterial sequences were attributable to 30 phyla, 77 classes, 183 orders, 269 families, and 439 genera. Actinobacteriota were the most abundant phylum, comprising approximately 42.3% of the total bacterial reads, followed by Proteobacteria (35.2% of reads) and Gemmatimonadota (5.1% of reads) (Supplementary Figure S1). The most abundant bacterial genera detected in all samples were *Gaiella* (4.4%), *KD4-96* (2.5%), *Microlunatus* (1.9%), and *Solirubrobacter* (1.8%). Bacterial richness ranged from 910 to 1616 ASVs and differed only between bare soil and the F7 rhizosphere upon nitrate treatment (Figure 4a). Fungal sequences were assigned to 10 phyla, 35 classes, 87 orders, 151 families, and 189 genera. Ascomycota (82.8% of reads) was the dominant fungal phylum, followed by Basidiomycota (11.0% of reads) (Supplementary Figure S2). The other fungal phyla accounted for less than 1.0% each. *Penicillium* and *Fusarium* were the most abundant genera identified in this study, accounting for 9.2 and 6.3% of the total fungal reads, respectively. Fungal richness ranged from 360 to 593 ASVs, and no differences were observed between treatments (Figure 4b).

### 2.4. Effect of Plant Presence and Nitrogen Fertilization on Bacterial and Fungal Communities

First, the extent to which the microbial community inhabiting the soil used in the experiment was shaped by nitrogen fertilization and the sown maize plants was investigated. PERMANOVA revealed that N fertilization and plant presence significantly affected the bacterial and fungal assemblage in the collected soil samples (Table 3). Specifically, these two experimental factors accounted each for about 10.0% of the variation in the bacterial community, while for the fungal population, plant presence and nitrogen fertilization captured about 9.0 and 8.0% of variance, respectively. No interaction was found between these two experimental variables in terms of community assembly for both microbial groups (Table 3).

**Figure 5:**
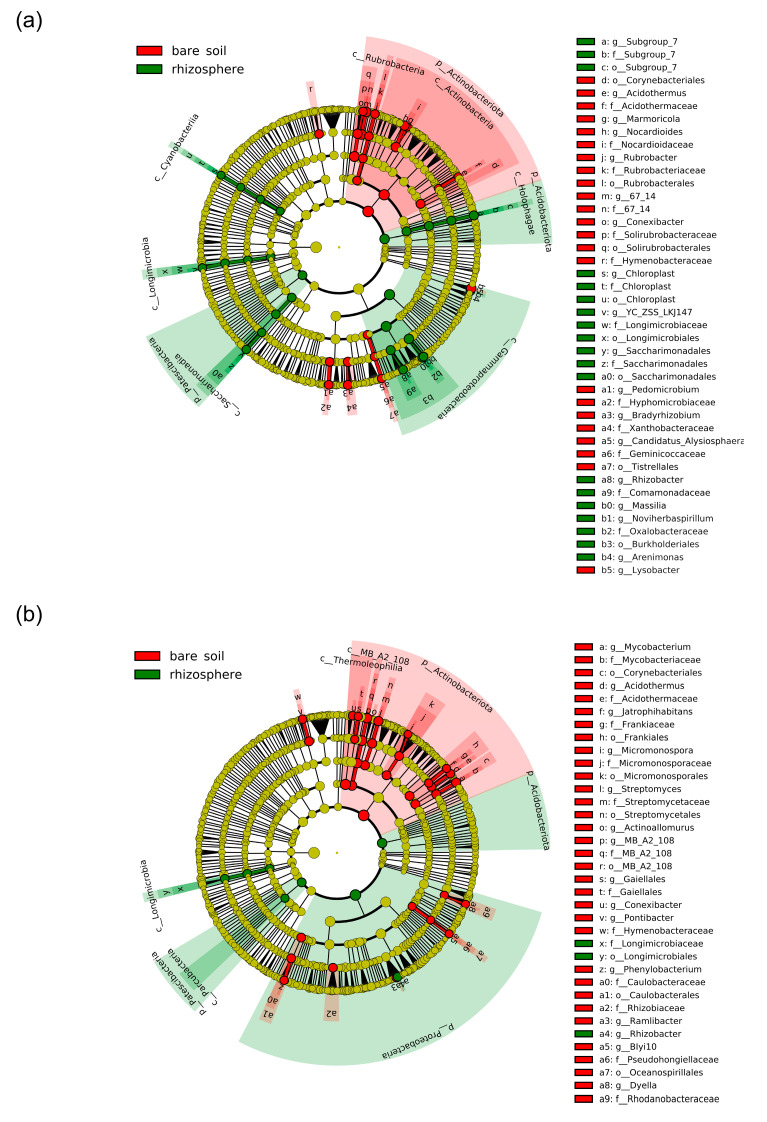
Cladogram illustrating the taxonomic groups explaining the most variation among the bacterial taxa between the bare soil and the rhizosphere samples detected in the (**a**) ammonium and (**b**) nitrate treatments. Each ring represents a taxonomic level, with phylum (p_), class (c_), order (o_), family (f_), and genus (g_) emanating from the center to the periphery. Each circle is a taxonomic unit found in the dataset, with circles or nodes shown in colors (other than yellow) indicating where a taxon was significantly more abundant.

**Figure 6:**
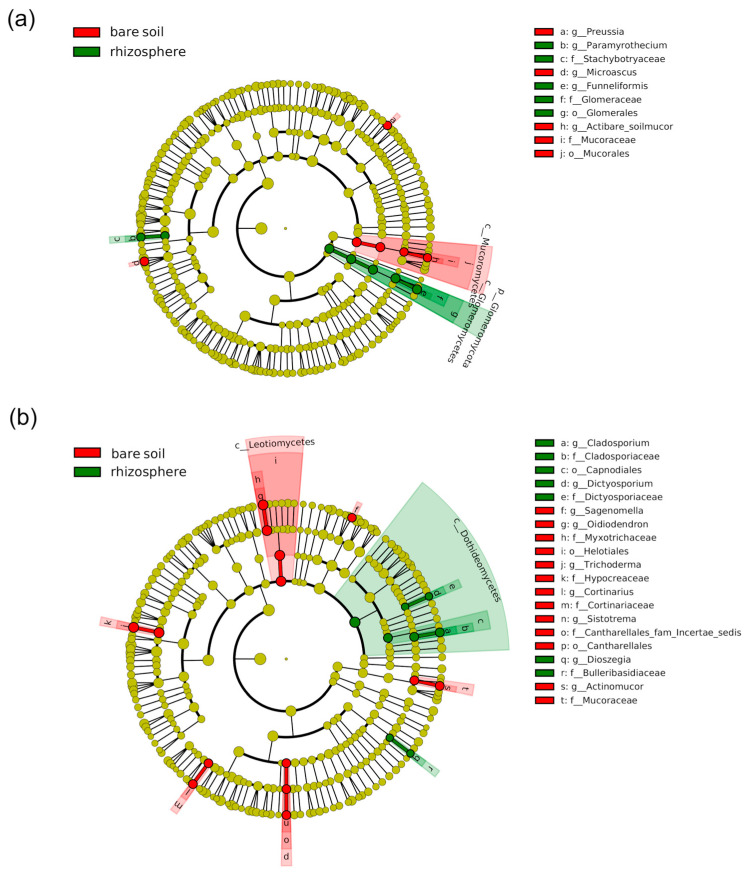
Cladogram illustrating the taxonomic groups explaining the most variation among the fungal taxa between the bare soil and the rhizosphere samples detected in the (**a**) ammonium and (**b**) nitrate treatments. Each ring represents a taxonomic level, with phylum (p_), class (c_), order (o_), family (f_), and genus (g_) emanating from the center to the periphery. Each circle is a taxonomic unit found in the dataset, with circles or nodes shown in colors (other than yellow) indicating where a taxon was significantly more abundant.

Considering the microbial community assemblage within each N treatment, a more pronounced plant effect was observed in the nitrate treatment compared to the ammonium treatment (Figure 4c–f; Tables S2 and S3). The bacterial populations were generally more affected by N form and plant presence than fungal communities. LEfSe analysis confirmed these observations, identifying nearly twice as many bacterial biomarker taxa as fungi in both N treatments (Figure 5 and Figure 6). More importantly, these compositional shifts observed in both nitrogen treatments were strongly phylogenetically clustered, as entire bacterial and fungal phyla responded markedly to the presence of plants. Indeed, an enrichment of taxa affiliated to the bacterial phyla Acidobacteria, Proteobacteria, and Patescibacteria, as well as to the class Longimicrobia (Gemmatimonadota phylum), was observed in all rhizosphere samples of maize (Figure 5). In contrast, the bare fertilized soil showed an increase in the abundance of bacteria from the Actinobacteriota phylum. At a finer taxonomic level, an increase in the abundance of genera associated to Proteobacteriaw was detected, such as *Arenimonas*, *Rhizobacter* and *Noviherbaspirillum*, and to the genus *Saccharimonadales* (Patescibacteria phylum) in the bacterial community of the rhizosphere of maize. Intriguingly, many Actinobacteriota genera were differentially enriched in the bare soils compared to the maize rhizosphere as a function of N fertilization, such as *Nocardioides*, *Conexibacte Mycobacterium*, *Jatrophhabitans*, *Micromonospora*, *Streptomyces,* and *Gaillelales* (Figure 5). It is also noteworthy that bacterial taxa involved in the nitrogen cycle were more abundant in fertilized bare soil samples than in the rhizosphere ones. This was the case for bacteria affiliated with the families *Frankiaceae* and *Nitrosospira,* which were found to be biomarker taxa for nitrate- and ammonium-fertilized bare soils, respectively.

The fungal communities also showed a phylogenetic response to the presence of plants, and it was mainly dependent on nitrogen fertilization (Figure 4). This was true for Glomeromycota fungi, as they were more abundant in the ammonium maize rhizosphere than in the corresponding bare soil (Figure 4), which however, was enriched in Mucoromycota taxa. The fungal community associated with the rhizosphere of maize in nitrate-fertilized soil was characterized by a higher proportion of taxa belonging to the Ascomycota class Dothideomycetes, whereas the bare soil associated with this treatment showed an enrichment of another Ascomycota class, the Leotiomycetes. At a finer taxonomic resolution, the presence of plants differentially affected the relative abundance of many Ascomycota genera depending on nitrogen fertilization. For instance, genera *Preussia*, *Pichia*, *Byssochlamys,* and *Microascus* were more abundant in the ammonium bare soil than in the corresponding maize rhizosphere, in which, however, the abundance of the genus *Paramyrothecium* increased. On the other hand, the rhizosphere fungal community was characterized by a higher proportion of the Ascomycota genera *Cladosporium* and *Dictysporium*, whereas an opposite trend was observed for the genera *Sagenomella*, *Oidiodendron,* and *Trichoderma*. Differences in the abundance of several genera belonging to the phylum Basidiomycota were also detected. Taxa belonging to the genera *Cortinarius* and *Sistotrema* were more abundant in the bare soil, while yeasts of the genus *Dioszegia* were biomarker taxa for the maize rhizosphere.

### 2.5. Differential Effect of N Nutrition and Maize Genotype on Bacterial and Fungal Rhizosphere Communities

Whether plant genotype and N treatment were associated with changes in microbial community structure was examined. These two predictors significantly influenced the bacterial assembly (Table 4). Maize genotype accounted for 10.6% of the variation in bacterial community structure, and nitrogen fertilization explained 13.1% of the variance. No significant interaction was found between these variables. In contrast, fungal community structure was not affected by the maize genotype, while N treatment accounted for approximatively 11.0% of variance. No interaction was found between these two terms for the fungal community assembly (Table 4).

Principal coordinate analysis (PCoA) of bacterial communities confirmed PERMANOVA results, as it clearly distinguished bacterial samples from the different nitrogen fertilizations along the first coordinate, with a separation between maize genotypes along the second coordinate (Figure S3). The fungal communities appeared to have a more dispersed pattern when projected onto the ordination plot than bacteria, although separation could be observed along the first coordinate between N treatments (Figure S4), mirroring PERMANOVA results as well (Table 4). 

**Figure 7:**
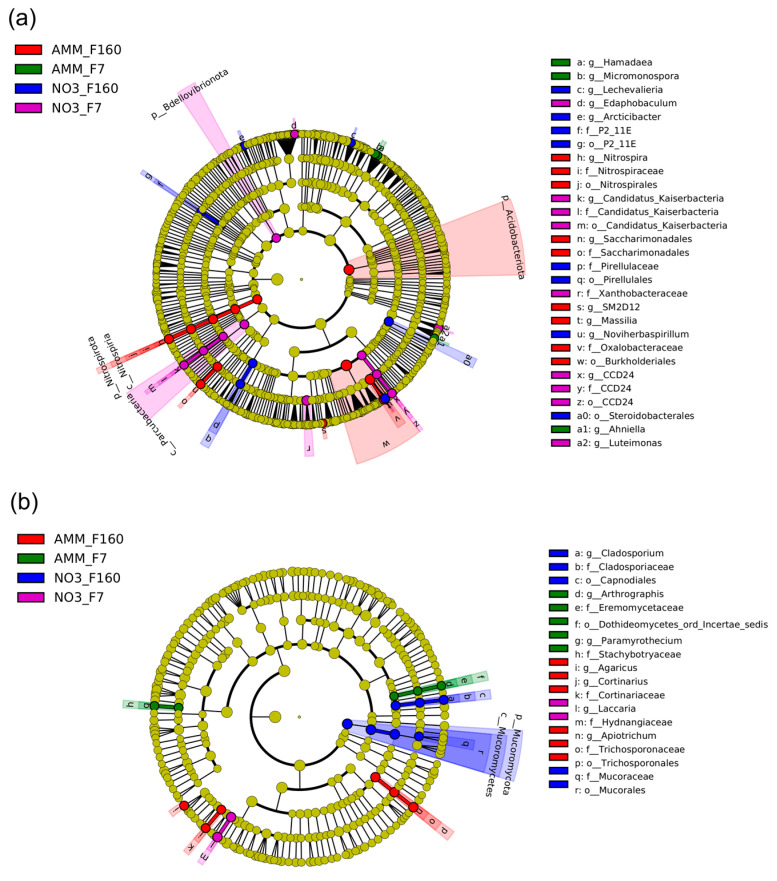
Cladogram illustrating the taxonomic groups explaining the most variation among the (**a**) bacterial and (**b**) fungal taxa between the different combinations of maize genotype (F160 or F7) and nitrogen treatment including ammonium (AMM) and nitrate (NIT). Each ring represents a taxonomic level, with phylum (p_), class (c_), order (o_), family (f_), and genus (g_) emanating from the center to the periphery. Each circle is a taxonomic unit found in the dataset, with circles or nodes shown in colors (other than yellow) indicating where a taxon was significantly more abundant.

The apparent differences in community structure between the bacterial populations associated with different maize genotypes and N fertilization treatments were mainly reflected in substantial shifts in the composition of the bacterial microbiota. In fact, LEfSe analysis identified several biomarker taxa for each maize genotype and mineral fertilization combination examined in this study (Figure 7a). For example, the phyla Acidobacteria and Nitrospirota and the order Burkholderiales (phylum Proteobacteria) were more abundant in the ammonium-fertilized rhizosphere of F160 than in the other genotype and N treatments. Similarly, bacterial genera with putative plant-promoting capability were enriched in the F160 rhizosphere under ammonium fertilization. This was the case of the genera *Massilia* and *Saccharimonadales*, which have been shown to have plant-growth-promoting properties. The F7 rhizosphere under ammonium nutrition also exhibited an increase in the abundance of putative plant-beneficial microbes, such as the genera *Hamadaea* and *Micromonospora*. Maize genotypes grown on nitrate-amended soil were enriched in a completely different set of bacterial taxa. Indeed, the F160 rhizosphere in the nitrate treatments showed an increase in abundance of the proteobacterial groups *Noviherbaspirillum* (genus) and the actinobacteria genus *Lechevaliera*. The rhizosphere of the maize genotype F7 grown under nitrate nutrition a displayed higher abundance of bacterial taxa associated with the Candidatus Kaiserbacteria group, the phyla Bdellovibrionota, the proteobacterial family *Xanthobacteraceae,* and the genus *Luteimonas*.

In contrast to the bacterial communities, only a handful of fungal taxa were found to be biomarkers of a particular maize genotype and N fertilization (Figure 7b), confirming the PERMANOVA and ordination plot analyses (Table 4, Figure S4). However, fungal taxa associated to the phylum Mucoromycetes were significantly more abundant in the rhizosphere of F160 fertilized with nitrate. In particular, the genus *Metarhizium* (Ascomycota), which encompasses entomopathogenic fungi in the *Clavicipitaceae* family, was found as a biomarker for this combination of N form and maize genotype. On the contrary, the F160 rhizosphere in the ammonium treatment was significantly enriched by several Basidiomycota genera, such as *Cortinarius*, *Agaricus,* and *Inocybe*, with the latter including several species with mycorrhizal capability. Taxa belonging to the *Laccaria* genus, another Basidiomycota genus with mycorrhizal capability, were found to be more abundant in the F7 rhizosphere under nitrate fertilization. On the other hand, fungal members affiliated with the Ascomycota genus *Paramyrothecium*, which contains species causing leaf spot, leaf blight, and stem and crown canker on many commercial crops worldwide [32,33], were instead found as biomarkers for the F7 rhizosphere community in the ammonium treatment.

Lastly, the contribution of the measured edaphic properties and plant traits on the microbiota assembly was assessed. RHL was found to be significantly correlated with bacterial community structure along with leaf P, Mn, K, and N concentrations (Table S4). Similarly, the fungal microbiota was correlated with leaf P, N, and K concentrations (Table S4).

## 3. Discussion

This study investigated how maize genotype and the chemical form of nitrogen nutrition influence P acquisition and whether the assembly of the rhizosphere microbiota is already affected in juvenile maize plants. Phosphorous is essential for plant growth and reproduction, playing a key role in cellular energy transfer, respiration, and photosynthesis, as well as being an essential structural component of nucleic acids and of many coenzymes, phosphoproteins, and phospholipids. However, the world’s soils are currently being depleted of P in spite of high chemical fertilizer input [34]. An adequate supply of P is essential during early maize growth, since early season limitations in P availability can result in severe restrictions on grain yield [35]. To test whether such an assumption is valid for F160 and F7 genotypes, the effect of low P on several plant fitness parameters was investigated in comparison to adequate P fertilization levels. Besides the expected P deficiency in shoots of maize plants grown under P-limited conditions, a differential response of maize plants to the low P availability was observed depending on genotype and chemical nitrogen form. Consequently, PUE and PUtE were most significantly affected by the P level, while PAE and PUtE were strongly affected by the chemical N form, with rather small effects of interactions between these parameters (Table 1). Furthermore, the modern variety F160 showed significant differences in shoot biomass when grown with sufficient P and ammonium fertilization, compared with maize plants grown with nitrate, but no differences were seen with limited P (Figure 1a). Likewise, while both maize genotypes grown in low-P soil showed the expected increase in relative root biomass [36,37], this was not significant and only a trend for F7 plants under nitrate nutrition. Although an increase in maize root elongation, density, and branching under P deficiency is widely reported [38,39], the present results support the crucial role of N nutrition and maize genotype on the mechanisms by which P deficiency affects root and shoot growth.

In the light of distinct effects of chemical nitrogen form on root traits, a multidisciplinary approach was employed to disentangle the effects of maize genotype and N form on P acquisition and on rhizosphere microbiota assemblage of juvenile maize plants in P-limited soil. As expected, rhizosphere soil pH was significantly reduced by ammonium (Table 2), regardless of the maize genotype. It is well-known that the use of ammonium as fertilizer leads to decreases in soil pH in the short and long term [40,41]. This soil acidification promoted by ammonium is frequently coupled with an improved P uptake from plants [3,42]. Although this was apparent in both genotypes (Figure 1c and Figure 2a), this had little or even negligible effect on the shoot biomass (Figure 1a). The acquisition of several other nutrients (K, Mn, Zn), however, was also affected by the P level and eventually by the N form (Figure 1d–g and Figure 2c–e). The positive effect of ammonium fertilization on P acquisition that is associated with rhizosphere acidification is linked with two important processes: (i) increase in the H_2_PO_4_^-^/HPO_4_^2-^ ratio and therefore increase in the dihydrogen phosphate ion that is preferentially absorbed by roots [43] and (ii) an increase the solubilization of Ca-phosphates [44]. The obtained findings are in line with the positive effect of ammonium fertilization on P acquisition, as significantly higher P concentrations in the maize shoots were found under ammonium than with nitrate fertilization, irrespective of the genotype (Figure 1c). This was seen both at sufficient P in the soil, as well as with low P availability (Figure 1c). Consequently, a significant increase in shoot PAE was observed in all the maize plants under ammonium nutrition, especially in the P-responsive genotype F7 (Figure 3).

Analysis of the microbial dataset revealed distinct bacterial (Figure 4 and Figure 5) and fungal (Figure 4 and Figure 6) communities associated with the maize rhizosphere with the distinct chemical N forms. It was observed that plant genotype and nitrogen supply strongly influenced microbial composition (Figure 4). Indeed, distinct microbial communities characterized each combination of these two experimental factors, with bacterial microbiota more susceptible than fungal populations (Figure 4).

Under P limitation, several plant attributes varied considerably across maize genotypes and N forms, such as root diameters and root hair length. These plant traits have been reported to differ among genotypes [45,46], and they play a crucial role in defining the nutrient absorption capacity of roots [47]. Root mycorrhizal colonization represents another key root functional trait involved in P acquisition [19,48]. Plants trade carbon with AMF in exchange for nutrients, mainly phosphorus and nitrogen [49,50,51], but also sulphur, potassium, calcium, iron, copper, and zinc [52,53,54]. The results revealed that ammonium fertilization increased AMF root colonization, especially in the F7 genotype (Table 2). Since AMF prefer to take up ammonium rather than nitrate from the soil [55], and since the N source at the symbiosis interface is mainly ammonium [56], a stimulatory effect of ammonium on mycorrhizal growth can be assumed. This could explain the higher AMF root colonization found in maize plants under ammonium nutrition, and it may be related to the positive effect of ammonium on P acquisition observed in the present study. Interestingly, the F160 genotype showed the typically inverse correlation between root hairs and mycorrhiza colonization, corroborating the general trade-off between root hairs and mycorrhizal symbiosis, in which plant species and genotypes with long and dense root hairs rely less on mycorrhizal fungi for P acquisition [15,57,58]. The results from the fungal microbial community composition confirmed the trend observed for root mycorrhizal colonization (Figure 6). Indeed, the maize rhizosphere under ammonium fertilization always harbored higher Glomeromycota abundance compared to nitrate nutrition, in particular in the F7 genotype (Figure 6 and Figure 7). These findings further support the positive effect of ammonium on mycorrhizal root colonization. As expected, the bare soil showed the lowest proportion in AMF affiliated taxa (Figure 6), corroborating the Glomeromycota lifestyle as obligate mutualistic symbionts [59]. 

Analysis of the microbial dataset further revealed distinct bacterial and fungal communities inhabiting the bare soil and maize rhizosphere (Figure 4, Figure 5 and Figure 6), outlining the determinant effect of maize growth on soil microbiota assembly after only four weeks of growth. Plants support and influence soil microbiota activity, mainly exuding organic compounds into the rhizosphere [60,61], and reciprocally, soil microbes engage in complex interactions with plants, influencing plant fitness [62,63,64]. In this study, considerable differences in microbial assembly between the bare soil and maize rhizosphere concerned taxa with a particular lifestyle, such as endophytes and plant-growth-promoting microbes (Figure 5 and Figure 6). The maize rhizosphere was characterized by a higher abundance of the genus *Saccharimonadia*, which encompasses taxa with putative plant-beneficial features, such as improving nitrogen uptake efficiency and promoting nutrient conversion [65,66]. Bacteria affiliated with the genus *Rhizobacter* are well-known plant-growth-promoting rhizobacteria [67,68,69], and they were found to be more abundant in the maize rhizosphere than in bare soil (Figure 4). A similar trend was observed by bacterial members associated with *Arenimonas*. *Arenimonas* represent an interesting bacterial genus, as they have been recently reported as endophytes in maize [70,71] and primarily were detected during early stages of maize growth [72].

Differences in microbiota community assembly between the treatments were correlated with the significant changes in plant attributes, in particular leaf P, N, and Mn concentrations and RHL (Table S4). Indeed, these plant traits have been commonly described as important drivers in structuring the rhizosphere microbiota [73,74], and their alterations might have important consequences in microbiota assembly [75,76,77]. At the community level, the rhizosphere bacterial population under ammonium fertilization harbored several plant-beneficial taxa with the capability to improve plant nutrient uptake. For instance, ammonium fertilization enriched the F160 rhizosphere in bacteria affiliated with the genus *Massilia*, which are known to be active microbiome members of the cereal rhizosphere, especially at the early stage of plant growth [78,79]. These bacteria have been reported as having plant-growth-promoting capabilities through P-solubilizing ability and indole acetic acid and siderophore production [80,81,82]. Intriguingly, *Massilia* taxa have shown antagonism activity towards crop pathogens [83,84] and the capability to improve salinity tolerance in maize [85]. Likewise, putative plant-beneficial bacteria affiliated with the genus *Saccharimonadales* were also found to be biomarkers for the F160 rhizosphere under ammonium fertilization. *Saccharimonadales* are capable of enhancing phosphatase activity in the rhizosphere [66,86,87], thus representing important microbes in phosphorus uptake by the plant. Ammonium fertilization also increases the abundance of another important bacterial genus involved in P uptake, *Micromonospora*. Taxa within *Micromonospora* have been widely described as notable P-solubilizing bacteria, since their ability to solubilize P is related to the excretion of organic acids, phosphatases, and calcium chelator [88,89,90].

Nitrate nutrition also shaped the maize rhizosphere microbiota in both genotypes investigated. It is worth mentioning that nitrate fertilization enriched the F160 rhizosphere in taxa associated to the *Lechevalieria* genus, which have been described as endophytic bacteria with a plant-promoting capability, especially in wheat cultivations [91,92]. Interestingly, a significant increase in the abundance of the entomopathogenic fungal genus *Metarhizium* was also observed with this treatment. Members of this fungal group represent a promising bioinsecticide against larvae of the chrysomelid *Diabrotica virgifera virgifera* (also known as “the western corn rootworm”), one of the major North American and European maize pests [93,94,95]. The rhizosphere of F7 under nitrate fertilization was mainly enriched in bacterial groups that are involved in the N cycle. This was the case of taxa affiliated with the family *Xanthobacteriaceae*, which encompass important nitrogen-fixing bacteria. Moreover, bacteria belonging to the genus *Luteimonas*, which are involved in the denitrification process, were found to be a biomarker for the nitrate F7 rhizosphere, thus confirming that the use of nitrate as an amendment could increase the abundance of denitrifying bacteria [96,97,98].

## 4. Materials and Methods

### 4.1. Experimental Setup

The topsoil of a green area (48°42′44″ N, 9°12′30″ E) with the following edaphic properties was used for the experiment: pH (CaCl2) 6.89, C_org_ 1.79%, (CaCO3 30%) 32.2 (mg kg^−1^) of P extracted with calcium acetate lactate (CAL-P), 0.02% total N and 95 CAL-K. Soil was fertilized with 100 mg K (K_2_SO_4_), 100 mg Mg (MgSO_4_·7H_2_O), 2.6 mg Zn (ZnSO_4_ 7 H_2_O), and 1 mg Cu (CuSO_4_ ·5H_2_O) per kg of dry soil. Nitrogen was fertilized in the form of nitrate (KNO_3_) or ammonium (ammonium sulfate with 3,4-Dimethyl-1H-pyrazole phosphate as nitrification inhibitor; NovaTec Soluble 21 Combo Expert, Germany), with 200 mg N added per kg dry soil. Phosphorus was fertilized in the form of Ca(H_2_PO_4_)_2_-H_2_O at a rate of 8.8 mg P kg^−1^ to adjust a final P concentration (measured CAL-P plus fertilized P) of 40 mg kg^−1^ for the low-phosphorus (LP) treatment. As a control (Ctrl), a soil substrate was used that was fertilized with either nitrate or ammonium and the same nutrient levels as described above, except for P, which was fertilized with 107.8 mg P kg^−1^ as Ca(H_2_PO_4_)_2_. These controls were necessary to obtain a comparison between the stressed and non-stressed plants in terms of biomass reduction and nutrient concentrations of the shoots, which provide insights into the internal P_i_ stress level. 

The fertilized soil was then mixed with 30% (*w*/*w*) quartz sand to improve soil texture and sieved with a 5 mm mesh. Soil moisture was adjusted to 70% of the water-holding capacity of the soil and gravimetrically adjusted every two days.

The experiment was performed in a growth chamber at the University of Hohenheim, Stuttgart, Germany (48°42′44″ N, 9°12′30″ E). Maize seeds of genotypes F7 and F160 were surface-sterilized with 10% (*v*/*v*) H_2_O_2_ solution for 20 min and placed in a 10 mM CaSO_4_ solution overnight at 25 °C. The next day, seeds were placed between filter paper soaked in a 4 mM CaSO_4_ solution for 3 days to germinate. The germinated seeds were selected and carefully transferred into the soil–sand substrate. Plants were grown in half-cylinder rhizotrons with a height of 48 cm and a diameter of 10 cm. The open part of the half-cylinder was covered with a transparent plexiglass observation window and secured with tape. Each rhizotron was filled with 2.4 kg of soil–sand substrate. Rhizotrons were arranged in a randomized design with five biological replicates for each treatment in a climate chamber. Maize plants were harvested 4 weeks after germination. The temperature in the climate chamber was maintained at 25 °C during the day and 20 °C at night, humidity was set at 60%, and the photoperiod was 14 h light/10 h dark. The photosynthetic photon flux density was adjusted to 300–350 µmol m^−2^ s^−1^ by adapting the height of the table.

Four weeks after germination, the soil–sand substrate was intensively rooted. Therefore, the bare soil was harvested by repeating the experimental setup without plants, keeping all nutrient and climate chamber conditions identical. Three biological replicates were used to compare microbial communities between the bare fertilized soils and the plant rhizosphere.

### 4.2. Maize Genotype Used in the Study

In this study, two flint lines, namely F160 and F7, with different P responsiveness were selected according to their biomass performance under low (LP) and sufficient phosphorus (Ctrl). Both genotypes were selected in the breeding program at the University of Hohenheim, Stuttgart, Germany. F7 represents a phosphorus founder line responsive to phosphorus, while F160 represents a non-phosphorus-responsive elite line with lower juvenile dry weight under low-P conditions [16]. Detailed information on these two genotypes can be found in Supplementary Table S1.

### 4.3. Determination of Plant Fitness Parameters

Each rhizotron was opened under the Semi 200-C microscope (Carl Zeiss, Jena, Germany) to record root conditions and root hair length non-destructively along the root observation plane. The root hair length (RHL) within each rhizotron was calculated as the average length of 25 photographed root hairs using the image software Axio Vision 3.1 (Carl Zeiss, Germany). After the root hairs were photographed, each rhizotron was destructively sampled to collect multiple plant data. Plants were carefully removed from the rhizotrons, and bulk soil was removed by shaking the plants. The rhizosphere soil was then sampled with a brush. Roots were carefully washed with deionized water, weighted, and stored in 70% (*v*/*v*) ethanol. Roots images were obtained by using an Epson scanner with a resolution of 400 dpi (Epson Expression 1000 XL, Tokyo, Japan). Root length and average root diameter of the scanned images were evaluated using WinRHIZO software (Reagent Instruments, Canada). After scanning, young root tips were selected, cut into 1 cm segments and stored again in 70% (*v*/*v*) ethanol. These root segments were further used to study mycorrhization. 

To determine the shoot biomass, the maize shoots were oven-dried at 60 °C for 96 h and weighted. Then, they were ground into fine powder. About 250 mg of the ground shoot dry matter was incubated in 5 mL HNO_3,_ 4 mL H_2_O_2_, and 2 mL of distilled water in a microwave oven (MLS Maxi 44, Leutkirch im Allgäu, Germany) at a maximum of 210 °C and 1.4000 W for 65 min. This solution was adjusted to 25 mL and filtered through a filter paper with a mesh size of 90 µm. Shoot P was measured spectrophotometrically via orthophosphate determination after addition of molybdate vanadate reagent [99]. Mn and Zn in the shoot were determined using an atomic absorption spectrophotometer (iCE 3000, Thermo Fisher Scientific, Waltham, MA, USA). Shoot K was determined using a flame photometer (Elex 6361; Eppendorf, Germany). Total N and C in the maize shoot were determined by elemental analysis (Vario Max CNS, elementar Analysensysteme GmbH, Langenselbold, Germany). Rhizosphere pH was measured using 2.5 g of the collected rhizosphere soil diluted in 25 mL of 0.01 M CaCl_2_ and stirred for 1 h at room temperature. The soil solution was then allowed to settle for 30 min. The pH of the supernatant was measured using a pH-meter (FiveEasyTM FE20, Mettler Toledo AG, Switzerland).

### 4.4. Definition and Calculation of Phosphorus Use Efficiency

In the context of this study, phosphorous use efficiency (PUE) is formally defined as the ability to accumulate biomass per unit P fertilizer applied under certain P conditions [100]. PUE was calculated using the following formula:

$$\mathrm{PUE}\;\left({\mathrm{mg}\;P}^{-1}{\;P}_{\mathrm{substrate}}\right)=\frac{\mathrm{Shoot}\;\mathrm{dry}\;\mathrm{weight}}{P_{\mathrm{substrate}}}$$

where P_substrate_ is the amount of *P* in the fertilized soil–sand mixture (equal to the P fertilizer added per rhizotron plus the measured CAL-P in the soil).

Next, phosphorus acquisition efficiency (PAE) was calculated, which refers to the ability of the plant to take up P from the soil through the roots, while phosphorus utilization efficiency (PUtE) refers to the ability to produce biomass, or yield, with this uptaken *P*. They were calculated using the following formulas:

$$\mathrm{PAE}\left({\mathrm{mg}\;P\;\mathrm{mg}}^{-1}{\;P}_{\mathrm{substrate}\;}\right)=\frac{P_{\mathrm{content}}}{P_{\mathrm{substrate}}}$$

where P_content_ is the amount of P in the shoot.

$$\mathrm{PUtE}\;\left({g\;\mathrm{DW}\;\mathrm{mg}}^{-1}{\;P}_{\;}\right)=\frac{\mathrm{Shoot}\;\mathrm{dry}\;\mathrm{weight}}{P_{\mathrm{content}}}=\frac{1}{\left[P\right]}$$

where [P] is the P concentration in the shoot in units of mg g^−1^ shoot dry weight. It is important to note that young maize plants under limited P availability efficiently remobilize P from storage sources, and much of the growth success depends on this remobilization efficiency. However, the formalism with PUE, PAE, and PUtE still gives interesting insight into key components of P usage in these genotypes.

### 4.5. Root Mycorrhizal Colonization

To assess mycorrhizal colonization of maize roots, about 50 root segments (1 cm) from each plant were collected and stained according to the protocol of [101] with slight modifications. Briefly, roots were cleared twice with 10% KOH at 90 °C for 45 min, rinsed several times with tap water, and acidified in 2 M HCl for 2 min. Then, roots were stained with 5% ink-vinegar solution at 60 °C for 30 min. Next, the root pieces were soaked overnight in tap water at room temperature. The tap water was acidified with several drops of acetic acid to remove excess ink [102,103]. The next day, the root segments were again washed several times with acidified tap water. The root segments were stored in acidic tap water until root colonization was determined. Thirty randomly selected root segments from each plant were fixed on slides (10 per slide), and their arbuscular mycorrhizal fungi (AMF) colonization was determined under a bright-field light microscope (Axioskop2, Zeiss, Germany). For each root fragment, mycorrhizal colonization was estimated according to [104], which allowed a rapid assessment of the AMF colonization level and the intensity of the ear root segment. The percentage of root length colonized by AMF was calculated as follows:

$$M\;=\frac{0.95\times N5+0.7\times N4+0.3\times N3+0.05\times N2+0.01\times N1}{N_{\mathrm{total}}\times 100\%}$$

where N_x_ = number of root fragments in five different AMF colonization intensity classes: >90%, >50%, <50%, <10%, <1%. Each N_x_ is multiplied with the respective relative coefficient of each intensity class (0.95, 0.7, 0.3, 0.05, 0.01). N_total_ = number of root fragments examined under the microscope.

### 4.6. Microbiome Analysis

Microbial community characterization was performed on three of the five established replicates of each plant genotype and for each nitrogen fertilization combination. Total genomic DNA was extracted from collected soil samples using the DNeasy PowerLyzer PowerSoil Kit (Qiagen, Hilden, Germany). Total bacterial community was characterized using the universal primer pair 27F/536R targeting the V1-V3 region of the bacterial 16S rRNA gene [105,106]. The primer set ITS3/ITS4 was used to amplify the fungal internal transcribed spacer (ITS) rRNA region [107]. PCR amplifications were performed in triplicate with a total volume of 50 μL reaction mix containing 1 μL of soil DNA template, 25 μL GoTaq G2 Master Mix (Promega, Mannheim, Germany), and 1 μL of each primer (25 μM). The produced amplicons were sequenced on an Illumina MiSeq instrument with 2 × 300 base pair kits by Eurofins Genomics Europe Sequencing (Constanz, Germany). Demultiplexing was performed using Illumina bcl2fastq 2.17.1.14 software following clipping of barcode and sequencing adapters. The DADA2 pipeline was used to determine amplicon sequence variants (ASV) from the raw reads [108]. For bacterial sequences, forward and reverse reads were truncated at 270 bp and 140 bp, respectively. For fungal reads, forward and reverse reads were truncated at 260 bp and 160 bp, respectively. Alpha diversity metrics were calculated from the normalized sequence library, with a threshold of 25,000 reads for bacterial and 40,000 reads for the fungal dataset. Representative bacterial 16S sequences were classified using the naive Bayesian classifier [109] for Silva v138. Representative fungal sequences were classified using the naive Bayesian classifier [109] against Unite 8.3 reference database [110]. All the raw sequences were submitted to the European Nucleotide Archive (study accession numbers PRJEB53850 for bacterial and PRJEB53851 for fungal data).

### 4.7. Statistical Analyses

The statistical analysis was performed using R version 4.1.2 (R Foundation for statistical computing, Vienna, Austria) [111]. To statistically evaluate the data, several analyses of variance were performed. To analyze P efficiencies, a three-way analysis of variance (ANOVA) was conducted with genotype, P treatment, N treatment, and their interaction as modelled effects. Two two-way ANOVA with either N fertilizer form, P-treatment and their interaction or genotype, N-treatment, and their interaction were performed to investigate P efficiencies such as PUtE and PAE and shoot and root biomass responses. One-way ANOVA was used to determine the effect of P treatment on shoot nutrient concentrations. Normality and variance homogeneity of the dataset were tested prior to ANOVA. A log10 transformation was applied to all variables that did not meet the parametric assumptions. Comparison of means was calculated using the Tukey HSD test at *p* < 0.05.

Differences in bacterial and fungal ASV richness were compared by ANOVA followed by Tukey’s HSD post hoc test. Microbial community structure was calculated using Bray–Curtis distance based on the relative abundance data with Hellinger transformation [112]. Permutational multivariate analysis of variances (PERMANOVA) based on the Bray–Curtis dissimilarity index was performed to analyze the effects of plant presence, application of different N forms, and maize genotypes on bacterial and fungal community structure, using 999 permutations for each test. Principal coordinates analysis (PCoA) based on the Bray–Curtis dissimilarity index was used to visualize the distribution of microbial community patterns. Linear discriminant analysis effect size (LEfSe) [113] was applied to identify biomarker taxa explaining differences between the microbiota in the different combinations of experimental variables.

The soil and plant properties were fitted to the nonmetric multidimensional scaling ordination using the “*envfit*” function in the “vegan” package of R, and goodness-of-fit statistics (R^2^) were calculated with *p*-values based on 999 permutations.

## 5. Conclusions

Overall, the presented findings demonstrated the key role of the chemical N form in mineral fertilizers, as well as genotype, in nutrient acquisition and microbiota assembly in the maize rhizosphere. As hypothesized, ammonium was shown to enhance maize P acquisition, but only in genotype F7 and not in F160. This was related to the decrease in pH of the rhizosphere and the accumulation of certain microbes associated with plant roots that play an important role in phosphorus uptake, such as AMF and phosphate-solubilizing bacteria. The fact that these findings were only partially observed in F160 suggests that the genotype plays an important role in how a particular N form affects P uptake. This highlights the importance of studying multiple genotypes when investigating plant response to P limitation. In addition, the effects of nitrogen fertilization on P uptake should be tested in other genotypes to better understand the role of N form in influencing P acquisition via changes in root morphologies and rhizosphere microbiomes. Along these lines, this study represents a fundamental starting point to understand how chemical nitrogen fertilizers can be used as a tool to select beneficial microbial communities associated with phosphorus uptake and fitness of maize plants in a future limited P scenario.

## Figures and Tables

**Table 1:** Effect of genotype, P treatment, N treatment, and their interaction on several P efficiencies associated to young maize plants (*n* = 5) calculated by ANOVA.

Source of Variation (Treatment)	PUE			PUtE			PAE		
	df	F	p	df	F	p	df	F	p
Genotype (G)	1	7.2	**0.0114**	1	5.2	**0.0292**	1	0.6	0.4426
P Treatment (P)	1	65.1	**<0.001**	1	45.0	**<0.001**	1	28.6	**0.0221**
N Treatment (N)	1	6.2	**0.0183**	1	28.6	**<0.001**	1	21.1	**<0.001**
G × P	1	0.4	0.5531	1	6.4	**0.0170**	1	3.1	0.0903
G × N	1	0.2	0.6972	1	1.0	0.3235	1	0.7	0.4210
P ×N	1	1.4	0.2516	1	1.1	0.3145	1	1.9	0.1757
G × N	1	5.7	**0.0232**	1	2.9	0.0988	1	0.7	0.3964

PUE = phosphorus use efficiency; PUtE = phosphorus utilization efficiency; PAE = phosphorus acquisition efficiency; df = degrees of freedom; F = F value; p = probability value. Significant probability values less than 0.05 are indicated in bold.

**Table 2:** Plant and soil properties measured in F7 and F160 maize plants under different nitrogen treatment.

Genotype	F7		F160	
N Treatment	NO3	AMM	NO3	AMM
Root Traits				
Root/Shoot	0.47 (0.12) A	0.33 (0.05) A	0.47 (0.07) A	0.43 (0.07) A
Rhizosphere pH	7.15 (0.07) AB	7.02 (0.04) B	7.27 (0.04) A	7.04 (0.03) B
M	0.09 (0.02) B	0.15 (0.01) B	0.15 (0.04) AB	0.17 (0.04) A
TRL [m]	34.1 (8.3) A	29.5 (1.7) A	35.4 (2.6) A	28.4 (2.4) A
SRL [m g^−1^]	64.4 (23) A	43 (5.7) A	45.7 (4.0) A	49.5 (9.4) A
RD [mm]	0.35 (0.01) B	0.38 (0.003) A	0.31 (0.008) C	0.33 (0.003) BC
FRL [m]	27.2 (6.8) A	22.4 (1.4) A	29.5 (2.3) A	23.2 (1.9) A
RHL [mm]	0.8 (0.03) AB	0.82 (0.02) AB	0.9 (0.03) A	0.74 (0.03) B

Data represent mean values of five biological replicates. Standard error of the means is indicated in parentheses. Different letters indicate significant differences at *p* < 0.05 according to Tukey’s HSD. M = percentage of root length colonized by AMF; m = mycorrhization intensity of mycorrhizal root fragments; TRL = total root length; SRL = specific root length; RD = root diameter; FRL = fine root length; and RHL: root hair length.

**Table 3:** The relative importance of plant presence and nitrogen treatment for the bacterial and fungal community structure associated with maize rhizosphere.

	Bacteria				Fungi	
Parameter	F	R^2^	p	F	R^2^	p
Plant (PL)	1.949	0.102	0.001	1.598	0.089	0.001
N treatment (N)	1.964	0.103	0.001	1.402	0.078	0.004
PL × N	1.179	0.061	0.133	0.969	0.054	0.541

F = F value; R^2^ = coefficient of determination; p = probability value.

**Table 4:** The relative importance of genotype and nitrogen treatment for the bacterial and fungal community structure associated with maize rhizosphere.

	Bacteria			Fungi		
Parameter	F	R^2^	p	F	R^2^	p
Genotype (G)	1.244	0.106	0.028	0.973	0.087	0.631
N treatment (N)	1.526	0.131	0.001	1.236	0.111	0.007
G × N	1.017	0.086	0.385	0.968	0.088	0.674

F = F value; R^2^ = coefficient of determination; p = probability value.

## Data Availability

All the raw sequences were submitted to the European Nucleotide Archive (study accession numbers PRJEB53850 for bacterial and PRJEB53851 for fungal data).

## Supplementary Materials

The following supporting information can be downloaded at: https://www.mdpi.com/article/10.3390/plants12030544/s1, Figure S1: Relative abundances of the phyla detected within the bacterial dataset; Figure S2: Relative abundances of the phyla detected within the fungal dataset; Figure S3: Principal coordinates analysis of the bacterial communities associated with the rhizosphere of the two maize genotypes in the different nitrogen treatments; Figure S4: Principal coordinates analysis of the fungal communities associated with the rhizosphere of the two maize genotypes in the different nitrogen treatments; Table S1: Information on the maize genotypes used in this study; Table S2: The relative importance of plant presence for the bacterial and fungal community structure associated with ammonium fertilization; Table S3: The relative importance of plant presence for the bacterial and fungal community structure associated with nitrate fertilization; Table S4: Goodness-of-fit statistics (R2) of environmental variables fitted to the nonmetric multidimensional scaling ordination of fungal and bacterial community structure in maize rhizosphere.
