# Supplementary material for: Nitrogen Fertilizer Type and Genotype as Drivers of P Acquisition and Rhizosphere Microbiota Assembly in Juvenile Maize Plants
Source: Plants (Basel). 2023 Jan 25;12(3):544. doi: 10.3390/plants12030544 (PMC9919524; doi:10.3390/plants12030544)
Supplement: Supplementary file 1 [file plants-12-00544-s001.zip › plants-2160568-supplementary.pdf]

plants

Supplementary Materials

Table S1. Information on the maize genotypes used in this study.

Genotype	Date of release	Background
F160	2015	Elite flint line
F7	1954	Founder line; Originated from landrace fromLacaune (France)

Copyright: © 2023 by the authors. Licensee MDPI, Basel, Switzerland. This article is an open access article distributed under the terms and conditions of the Creative Commons Attribution (CC BY) license (https://creativecommons.org/licenses/by/4.0/).

Table S2. The relative importance of plant presence for the bacterial and fungal community structure associated with ammonium fertilization.

	**Bacteria**				**Fungi**			
**Parameter**	df	F	R^2	P	df	F	R^2	P
**Plant**	1	1.401	0.166	0.009	1	1.244	0.146	0.042

Table S3. The relative importance of plant presence for the bacterial and fungal community structure associated with nitrate fertilization.

	**Bacteria**				**Fungi**			
**Parameter**	df	F	R^2	P	df	F	R^2	P
**Plant**	1	1.711	0.196	0.008	1	1.312	0.159	0.029

Table S4. Goodness-of-fit statistics (R2) of environmental variables fitted to the nonmetric multidimensional scaling ordination of fungal and bacterial community structure in maize rhizosphere. Only significant variables are reported.

	Bacteria		Fungi	
**Parameter**	R^2	P	R^2	P
**P**	0.4672	**0.039**	0.4172	0.065
**Mn**	0.6606	**0.006**	0.0323	0.849
**K**	0.551	**0.026**	0.5509	**0.027**
**N**	0.6033	**0.016**	0.5823	**0.020**
**RHL**	0.4738	**0.043**	0.4434	**0.046**

Bold values indicates statistical significance.

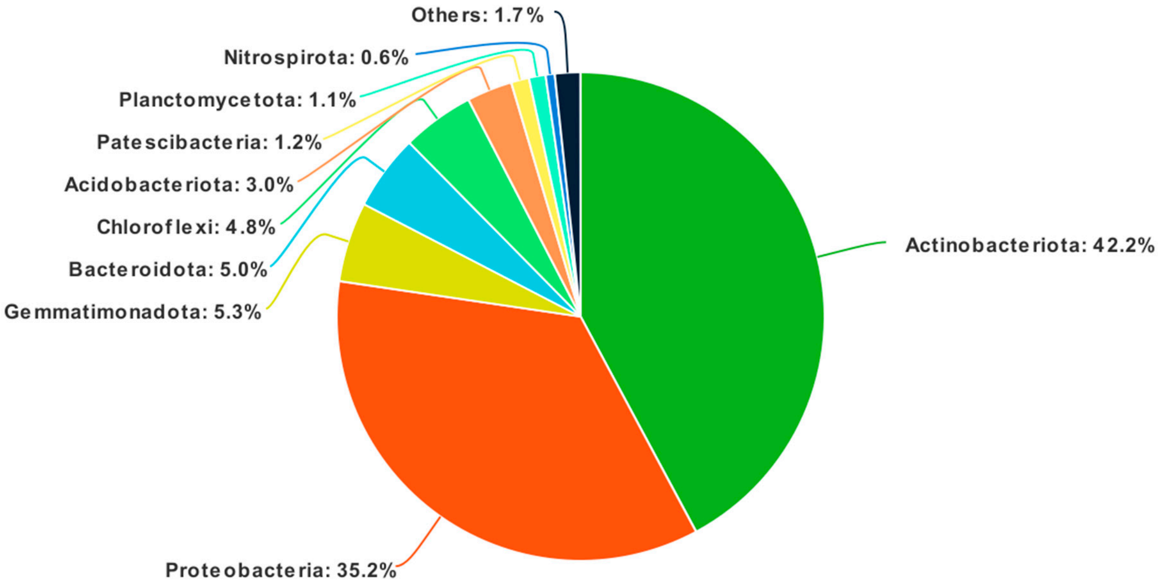

Figure S1. Relative abundances of the phyla detected within the bacterial dataset.

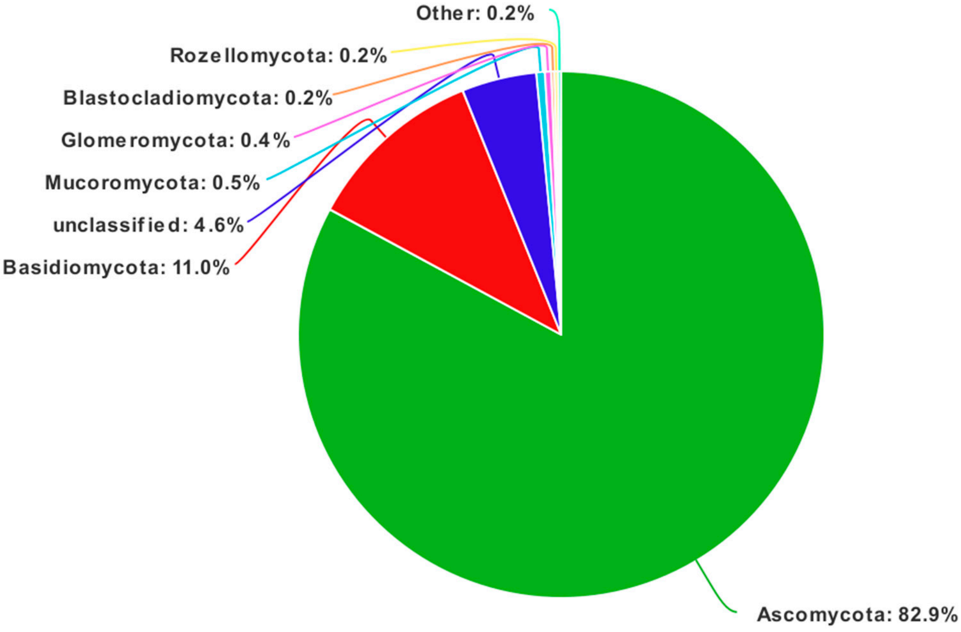

Figure S2. Relative abundances of the phyla detected within the fungal dataset.

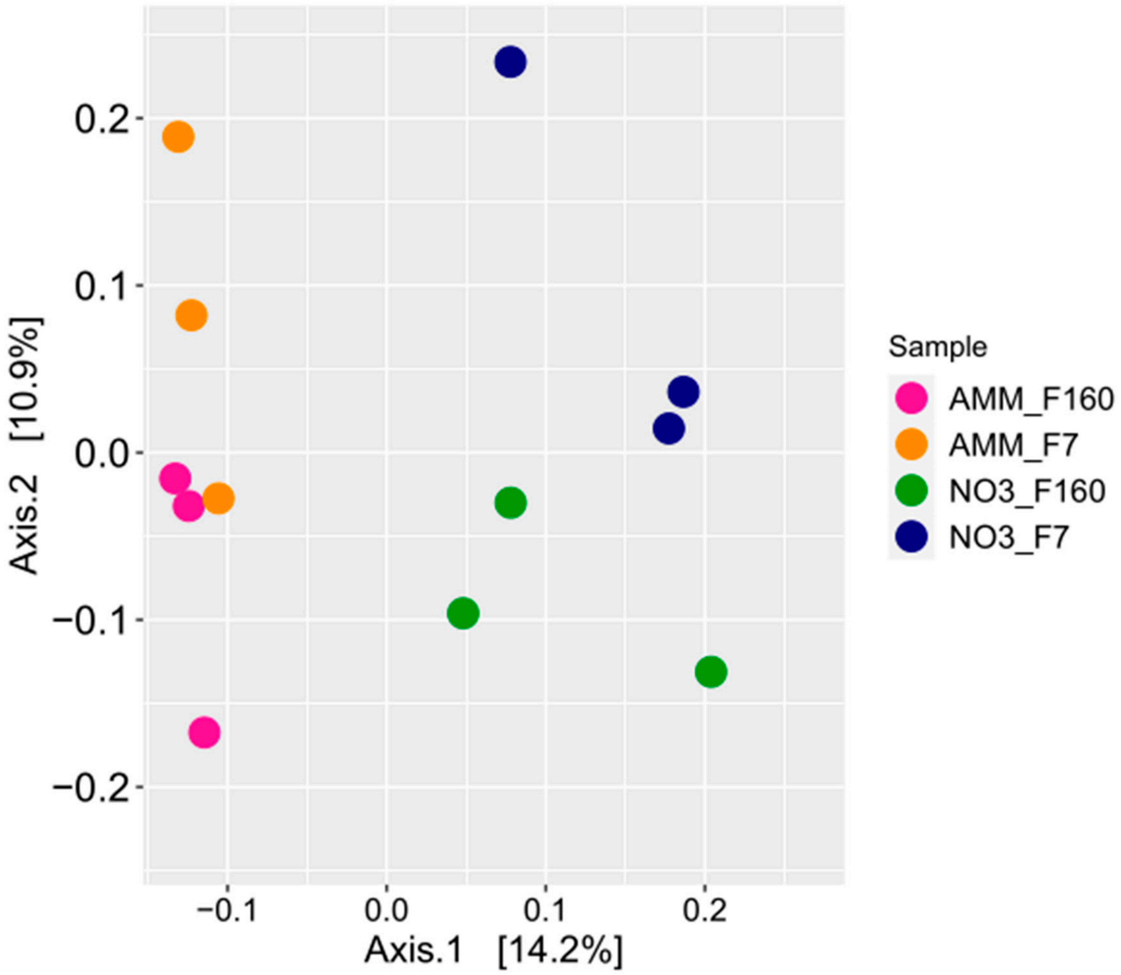

Figure S3. Principal coordinates analysis of the bacterial communities associated with the rhizosphere of the two maize genotypes in the different nitrogen treatments.

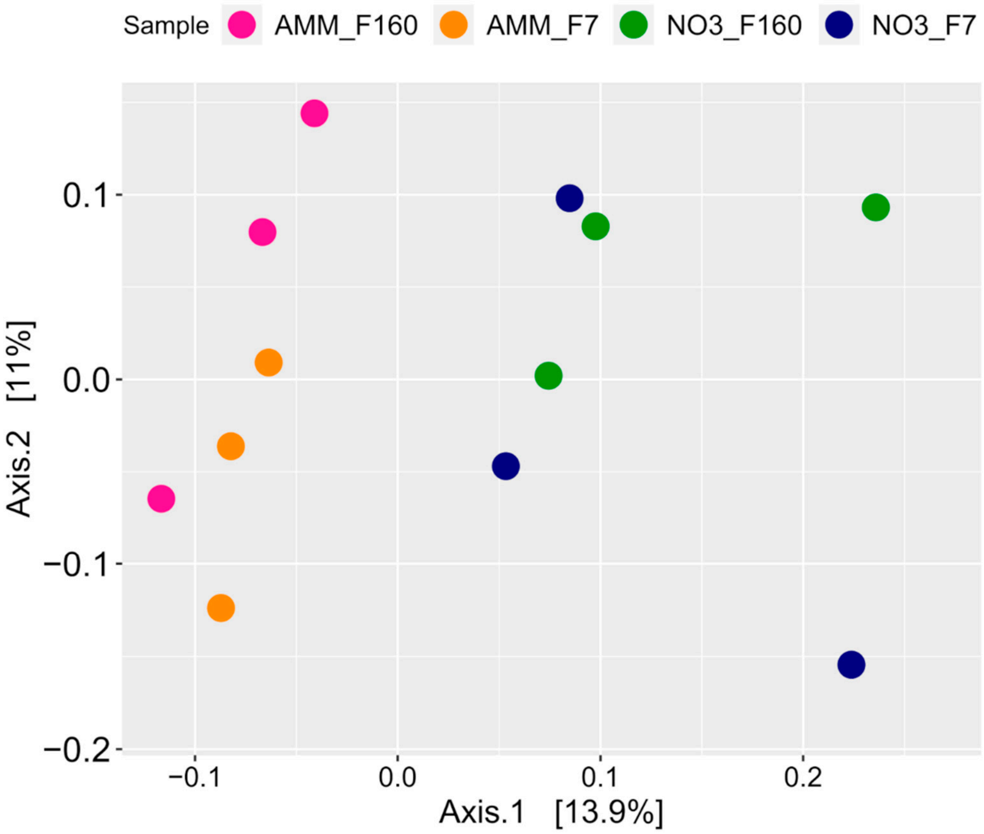

Figure S4. Principal coordinates analysis of the fungal communities associated with the rhizosphere of the two maize genotypes in the different nitrogen treatments.

Disclaimer/Publisher's Note: The statements, opinions and data contained in all publications are solely those of the individual author(s) and contributor(s) and not of MDPI and/or the editor(s). MDPI and/or the editor(s) disclaim responsibility for any injury to people or property resulting from any ideas, methods, instructions or products referred to in the content.
